# Long-Term Seasonal and Interannual Patterns of Marine Mammal Strandings in Subtropical Western South Atlantic

**DOI:** 10.1371/journal.pone.0146339

**Published:** 2016-01-27

**Authors:** Jonatas H. F. Prado, Paulo H. Mattos, Kleber G. Silva, Eduardo R. Secchi

**Affiliations:** 1 Instituto de Oceanografia, Laboratório de Ecologia e Conservação da Megafauna Marinha, Universidade Federal do Rio Grande, Rio Grande, Brazil; 2 Instituto de Oceanografia, Laboratório de Gerenciamento Costeiro, Universidade Federal do Rio Grande, Rio Grande, Brazil; 3 Núcleo de Educação e Monitoramento Ambiental, Rio Grande, Brazil; Institute of Marine Research, NORWAY

## Abstract

Understanding temporal patterns of marine mammal occurrence is useful for establishing conservation strategies. We used a 38 yr-long dataset spanning 1976 to 2013 to describe temporal patterns and trends in marine mammal strandings along a subtropical stretch of the east coast of South America. This region is influenced by a transitional zone between tropical and temperate waters and is considered an important fishing ground off Brazil. Generalized Additive Models were used to evaluate the temporal stranding patterns of the most frequently stranded species. Forty species were documented in 12,540 stranding events. Franciscana (n = 4,574), South American fur seal, (n = 3,419), South American sea lion (n = 2,049), bottlenose dolphins (n = 293) and subantarctic fur seal (n = 219) were the most frequently stranded marine mammals. The seasonality of strandings of franciscana and bottlenose dolphin coincided with periods of higher fishing effort and strandings of South American and subantarctic fur seals with post-reproductive dispersal. For South American sea lion the seasonality of strandings is associated with both fishing effort and post-reproductive dispersal. Some clear seasonal patterns were associated with occurrence of cold- (*e*.*g*. subantarctic fur seal) and warm-water (*e*.*g*. rough-toothed dolphin) species in winter and summer, respectively. Inter-annual increases in stranding rate were observed for franciscana and South American fur seal and these are likely related to increased fishing effort and population growth, respectively. For subantarctic fur seal the stranding rate showed a slight decline while for bottlenose dolphin it remained steady. No significant year to year variation in stranding rate was observed for South American sea lion. The slight decrease in frequency of temperate/polar marine mammals and the increased occurrence of subtropical/tropical species since the late 1990s might be associated with environmental changes linked to climate change. This long-term study indicates that temporal stranding patterns of marine mammals might be explained by either fishing-related or environmental factors.

## Introduction

Aerial and shipboard surveys improve knowledge of marine mammal distribution [1]. However, they are very expensive and data collection can be challenging as most marine mammals (especially cetaceans) are highly mobile and spend substantial time below the surface. Another means of determining marine mammal presence, and potentially relative abundance, is by monitoring strandings. Compared with aerial and shipboard surveys, monitoring strandings is inexpensive and logistically simple. Some of the biases associated with stranding data can be counteracted by using long time series of data collected systematically. This can reduce the effects caused by a small number of atypical strandings and it can allow the investigator to distinguish regular patterns from random variation [2,3]. In Hawaii, analyses based on both odontocete stranding events and at-sea surveys collected over 65 years revealed that stranding records provide reliable data on the occurrence of species and are good indicators of species composition [4]. In addition, systematic beach surveys have been critical for documenting and monitoring marine mammal mortality due to human activities such as fisheries (*e*.*g*. [5,6]) and to ‘natural’ die-offs (*e*.*g*. [7,8]).

The coast of Rio Grande do Sul (RS), southern Brazil, has been surveyed to record and collect marine mammal carcasses for almost four decades. During this long-term beach monitoring program, 40 species have been recorded (*e*.*g*. [9–18]). However, studies focusing on temporal variability in the stranding data have been limited to only a few species such as franciscana, *Pontoporia blainvillei*, [6,19], bottlenose dolphin, *Tursiops truncatus* [20], South American sea lion, *Otaria flavescens* and the South American fur seal, *Arctocephalus australis* [21], and have taken place over a much smaller timeframe.

The high diversity of marine mammal species in this region is probably associated with great variability in the environmental characteristics that exist on the continental shelf and beyond. Interaction between the wind-driven current and the western boundary currents (Brazil and Malvinas/Falkland) over the shelf produce a southward and offshore flow during summer and a northward and onshore flow during winter [22]. This seasonal reversal in the direction of flow over the shelf and the change from warm, nutrient-poor coastal water in summer to subantarctic, nutrient-rich water in winter have profound effects on biological productivity and ecosystem dynamics [23–25]. Furthermore, the high phytoplankton biomass during winter and spring has been related to nutrient supply from freshwater discharge of the La Plata River and Patos Lagoon Estuary [23,25]. The presence of both subantarctic waters and freshwater input, mainly in winter and spring, makes the continental shelf one of the most productive and important fishing area off Brazil [23,26,27]. In this context, the diversity and abundance of top predators, such as sharks, sea birds and marine mammals [28–32] (ECOMEGA unpubl. data), as well as the intensity of fishing effort, vary seasonally [26,31].

Bycatch in fisheries is one of the most significant threats to marine mammals [33,34]. In southern Brazil, mortality due to incidental entanglement in coastal gillnets is by far the greatest threat to the franciscana and to a small population of bottlenose dolphins [20,35,36]. For example, the annual mortality of franciscanas in gillnet fisheries in southern Brazil ranges from several hundreds to a few thousand individuals (*e*.*g*. [6,35–38]). Since the early 1980s coastal gillnet effort has increased in this region [27]. The mean net length of most of this fleet has increased fourfold since the mid 1990s [35,37,39]. Therefore, an increase in mortality of this coastal species would be expected. Although there is no estimate of trawl-related mortality of South American sea lions in southern Brazil, nearly 50 animals are killed annually in this fishery in the neighboring area of Uruguay where fishing effort is lower than in southern Brazil (*e*.*g*. [40]). According the regional agency for fishing policy [41] about 80 trawlers are operating along the RS coast, conducting around 500 annual fishing trips.

Inferring the causes of a marine mammal’s stranding is difficult as the number of beached carcasses depends on many underlying processes (*e*.*g*. at-sea mortality, buoyancy, drift, and detection probability) [6,42,43]. The absence of stranding does not imply that at-sea mortality has not occurred. During periods of strandings-unfavorable conditions the carcasses can be transported offshore or to areas where the detection probability is low (*e*.*g*. [44,45]). Yet another constraint is to attribute the cause of mortality. In the case of fishing-related mortality of marine mammal, only a few carcasses may present clear evidence of such interaction. Despite of those limitations, in southern Brazil, some studies were able to link strandings of coastal species to mortality in fishing gear (*e*.*g*. franciscana and bottlenose dolphin–[6,20,39]).

It is important to emphasize that stranding data collected during a long time period offer an unprecedented opportunity in detecting trends in fishing related mortality as well as changes in marine mammal communities attributed to variation in the physical-chemical and biological properties of the environment. For example, it is expected a higher frequency of tropical and temperate or polar species during warmer and colder periods, respectively. Therefore, the main objective of the present study was to describe temporal (seasonal and annual) patterns of marine mammal stranding based on the longest continuous time series of stranding data for the southwestern Atlantic Ocean. It is expected that the results can be used to design conservation strategies for the marine mammals, especially for those species that are most vulnerable to bycatch.

## Materials and Methods

### Study area

The coast of RS state is oriented northeasterly to southwesterly and comprises a 618km stretch of sandy beach. It is interrupted to the north by the Tramandaí Lagoon inlet and to the south by the Patos Lagoon Estuary inlet. The continental shelf is relatively flat and wide (100km in the north to 180km in the south) with a smooth slope (2m km^-1^) to the shelf break, which begins near the 150–200m isobath [46]. This region has a seasonally variable wind regime, with northeasterly winds dominating in summer and generating a southward flow of coastal waters, and southwesterly winds in winter, with coastal waters flowing northward [24]. The coastal area is influenced by Subantarctic Shelf Water transported northward by the Malvinas/Falkland Current (MFC) and Tropical Water and South Atlantic Central Water transported southward by the Brazil Current (BC) [24]. The western boundary of the Subtropical Convergence (confluence between the MFC and BC) is located mainly along the shelf break, and shows remarkable seasonal migration. The northern limit of the Subtropical Convergence fluctuates between 33°S in winter and 38°S in summer [47]. Besides the oceanic water influence, the discharge of large amounts of fresh water in the coastal zone from the La Plata River and the Patos Lagoon Estuary has a strong impact on shelf dynamics [24]. During austral winter (July-September) the river plume extends further than Santa Marta Cape (28°S) while in summer (January-March) it retracts to approximately 32°S. The seasonal wind field seems to be the main factor driving such a pattern [48]. In summer, northeasterly winds (upwelling-favorable) force the plume to its southernmost position and it occupies a large portion of the shelf due to offshore Ekman transport, while in winter the southwesterly winds (downwelling-favorable) displace the plume to the north, restricting it to a narrow strip along the coast [24]. This northward flow off southern Brazil has been referred as the Brazilian Coastal Currents [49].

**Fig 1:**
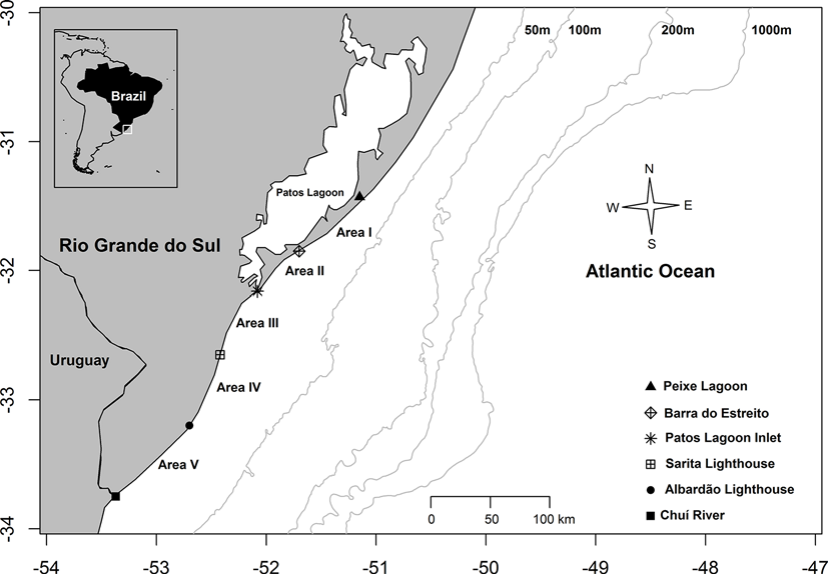
Study area. Area 1 = 87 km; Area 2 = 48 km; Area 3 = 63 km; Area 4 = 70; Area 5 = 87 km.

In the present study beach surveys were carried out along a 355 km stretch of coastline in central and southern RS, from Peixe Lagoon (31°26’S—51°09’W) to Chuí (33°45’S—53°22’W) at the border between Brazil and Uruguay. The study area was divided into five subareas: Area I (87 km long) located between the Peixe Lagoon and the Barra do Estreito (31°51’S—51°42’W); Area II (48 km long) located between the Barra do Estreito and the Patos Lagoon Estuary mouth (32°08’S—52°04’W); Area III (63 km long) located between the Patos Lagoon Estuary mouth (32°09’S—52°05’W) and the Sarita Lighthouse (32° 39’ S—52° 25’ W); Area IV (70 km long) located between the Sarita Lighthouse and the Albardão Lighthouse (33°12’S—52°42’W); and Area V (87 km long) located between the Albardão Lighthouse and Chuí (Fig 1).

### Data collection

In years before 1979 some opportunistic sampling was derived from 8 beach surveys (totaling 531km) and occasional notifications of strandings by locals. After that, records were obtained from systematic beach surveys from 1979 to 2013. During that period, occasional notifications were used only for the purpose of improving accuracy on the month of stranding.

A gap of systematic sampling occurred between 1988 and 1991, except for two surveys conducted during the summer of 1988. After the early 1990s, when weather conditions allowed, beach surveys were carried out fortnightly from Lagoa do Peixe to Chuí (see Results). The surveys were conducted by three research groups: 1) *Laboratório de Ecologia e Conservação da Megafauna Marinha*, Universidade Federal do Rio Grande–ECOMEGA/FURG (from 1976 to 1988 and 1992 to 2013); 2) *Museu Oceanográfico Prof*. *Eliezer de C*. *Rios*—MO/FURG (during the 1990s); 3) *Núcleo de Educação e Monitoramento Ambiental*—NEMA (from 1993 to 2013).

A four-wheel-drive vehicle was used with two to four observers scanning from the wash zone up to the base of the sand dunes (ranging from 30 to 60 m in width) at speeds of 60–70 km/h. The data collection protocol for stranded marine mammals was standardized including: date, geographical location, standard body length and decomposition state [50], as follows: 1 = alive; 2 = freshly dead; 3 = moderate decomposition; 4 = advanced decomposition; 5 = mummified or skeletal remains. In cases of records for which the decomposition state was not reported, the month of stranding was assumed to be the month of the survey. For those animals at decomposition state 5, we arbitrarily considered that the stranding had occurred 30 days earlier. Carcasses with pieces of nets attached to the body, lacerated and/or amputated fin or flipper (it is a common practice of fishermen to cut some appendage to remove dolphins from their nets), net marks and the presence of firearm bullets (as in the case of a few sea lions) were considered to have been killed due to fishery interactions. Survey data provided by MO/FURG and NEMA were only included in the database when the time between consecutive surveys carried out by ECOMEGA exceeded one month. Based on field observations and researchers’ experience, small carcasses (up to 2m long) can be missed if surveys are carried out in time intervals longer than one month. Since most carcasses were not removed from the beach, they were sprayed with color paint or labeled by all institutions to minimize chances of double counting. Sampling from stranded carcasses was made with permission issued by the Ministry of Environment, Brazil Government (License Sisbio # 16586–2), in compliance federal legislation.

### Data selection

Only dead animals were considered for the purpose of the analyses on temporal patterns of stranding. The stranded species were classified in three categories according to their frequency of occurrence: 1) frequent species (stranding frequency (SF) ≥0.02), 2) occasional species (SF between 0.02 and 0.0005), 3) rare species (SF ≤0.0005).

Each stranding was considered as a separate event, except for mass strandings, here defined as events where two or more individuals of the same species (excluding mother-calf pairs) were found within 10 km from one another and presented the same decomposition state within the range of 2 to 4 (i.e. it is generally not possible to determine with confidence the date of strandings of animals in decomposition state 5). These criteria were not applicable to pinnipeds and to franciscana, due to its very high fishing-related mortality.

As the high mortality of neonates of South American fur seal from reproductive colonies in Uruguay can mask stranding patterns of older animals when all strandings are combined, individuals with a total length of < 110cm were analyzed separately. Hereafter, individual fur seals <110cm and >110cm will be referred to as neonates and juveniles/adults, respectively.

### Data analyses

The number of stranded marine mammals per 100km of beach surveyed (hereafter referred to as stranding rate) was used to describe seasonal and inter-annual frequency distribution of strandings. Seasons were classified as: spring (October–December), summer (January–March), autumn (April–June) and winter (July–September).

Temporal stranding patterns of the frequent species were evaluated through Generalized Additive Model (GAM) [51]. This method is a natural choice when the relationship between the response and predictor variables is complex and not easily modeled by specific linear or non-linear functions. We used a log link function and a Negative Binomial error distribution to account for over-dispersion [42]. Year and month were used as the predictor variable and number of stranding as the response variable. To investigate the time series for seasonal variability of strandings, an interaction term of the two explanatory variables was included. As the beach survey effort (km of beach survey) was not evenly distributed across de years it was included in GAM as an offset. This term adjusted otherwise independent counts (number of stranded marine mammals) per kilometer of beach surveyed in which it was made (see [52]). The performance of three alternative models was assessed:

Model1:number of stranding=f(year)+f(month)+year*month+offset

Model2:number of stranding=f(year)+f(month)+offset

Model3:number of stranding=f(month)+offset

where *f* are smooth functions.

The best model was selected with the Akaike Information Criterion (AIC). Results from statistical models were validated using diagnostic plots. The GAM analysis was restricted to the period 1992–2013, as it represented the largest continuous data series from systematic surveys without interruption.

The inclusion of stranding records from areas in which survey effort was not evenly distributed across years can lead to incorrect interpretation of temporal patterns. Therefore, we used only the data from areas II and III (evenly distributed across years, see Results) for temporal analyses. GAMs were run with the package mgcv 1.4–1.

All analyses and visualizations were performed using the software R, version 3.1.2 [53].

## Results

From 1976 to 2013 the total cumulative distance covered as part of the beach surveys was *ca*. 118,178 km. Survey effort was more intensive in Areas II and III between 1979 and 1988. After 2002, effort increased and became more evenly distributed across areas (Table 1, S1 Fig).

**Table 1:** Total kilometer of beach surveyed and the number of times that beach survey was concluded in each area from 1976 to 2013. I = 84km; II = 51km; III = 63km; IV = 70km; V = 87km.

Year	I		II		III		IV		V	
	Total km	N° of time concluded	Total km	N° of time concluded	Total km	N° of time concluded	Total km	N° of time concluded	Total km	N° of time concluded
**1976–1979**	87	1	237	4	335.2	2	70	1	87	1
**1980–1988**	186	1	3,689.5	67	6,336.8	87	1,309	14	1,162	2
**1992–2001**	3,850.7	38	5,859.3	118	10,792.5	165	5,562.5	69	5,688.3	57
**2002–2013**	14,411.9	131	10,792.0	224	15,983.6	251	15,431.9	199	16,306.6	176
**Total**	18,535.3	171	20,577.8	413	33,448.1	505	22,373.4	283	23,243.9	236

A total of 12,540 marine mammals stranding events (779 live and 11,761 dead strandings), involving animals from 10 families and 40 species (species could not be determined for 1,232 events), were reported in the study area. Franciscana (n = 4,574), South American fur seal (n = 3,419), South American sea lion (n = 2,049), bottlenose dolphin (n = 293) and subantarctic fur seal, *Arctocephalus tropicalis*, (n = 219) were the most frequently reported species. The other 35 species accounted for 3% of the remaining marine mammal stranding events (Table 2). The majority of strandings involved a single individual. Only seven events were mass strandings (S1 Table). The decomposition state of stranded animals was determined for 57% (n = 7,186) of the records and of those, 31% (n = 2,276) and 22% (n = 1,628) were putrefied (Codes 4 and 5, respectively).

**Table 2:** The number of individual (inds) marine mammal strandings observed from 12,540 reported events (evts) in southern Brazil from 1976 to 2013. The total stranding events for each species is given as number and as percentage. Mean annual stranding rates are shown in the last column.

**Species**	Classification	Year										Dead(inds)	Dead(evts)	%	AnnualStranding rate
		1976–1979		1980s		1990s		2000s		2010–2013					
		Dead	Alive	Dead	Alive	Dead	Alive	Dead	Alive	Dead	Alive				mean (sd)
*Pontoporia blainvillei*	Frequent	171	1	602		518		2442		841		4574	4574	38.90	3.5826(3.6711)
*Arctocephalus australis*	Frequent	6	3	179	12	514	38	1429	265	1291	69	3419	3419	29.08	2.1831(1.9449)
*Otaria flavescens*	Frequent	27		310	4	509	2	808	16	395	3	2049	2049	17.43	1.7960(0.7853)
*Tursiops truncatus*	Frequent	20		57		44		113		59		293	293	2.49	0.2687(0.2246)
*Arctocephalus tropicalis*	Frequent			19	3	45	10	138	25	17	5	219	219	1.86	0.1885(0.3326)
*Eubalaena australis*	Occasional	1		6		18		10		7		42	42	0.36	0.0215(0.0442)
*Pseudorca crassidens*	Occasional			5		27		12		9		53	38	0.32	0.0249(0.0374)
*Globicephala melas*	Occasional			3		11		10		3		27	27	0.23	0.0173(0.0345)
*Steno bredanensis*	Occasional			1		7		5		11		24	21	0.18	0.0098(0.0196)
*Physeter macrocephalus*	Occasional	2		4		5		8		4		23	20	0.17	0.0236(0.0608)
*Balaenoptera acutorostrata*	Occasional			1		7		9		1		18	18	0.15	0.0128(0.0250)
*Delphinus delphis*	Occasional					4		7		3		14	14	0.12	0.0055(0.0122)
*Orcinus orca*	Occasional	1		1		7		4		1		14	14	0.12	0.0187(0.0850)
*Lagenodelphis hosei*	Occasional					9		2		3	1	14	13	0.11	0.0073(0.0196)
*Stenella frontalis*	Occasional					2		6		1		9	9	0.08	0.0048(0.0127)
*Megaptera novaeangliae*	Occasional			1		1		2		4		8	8	0.07	0.0034(0.0079)
*Kogia breviceps*	Occasional			3		1		2		1		7	7	0.06	0.0018(0.0064)
*Mironga leonina*	Rare			1	3	2	1	1	1	2	3	6	6	0.05	0.0062(0.0196)
*Phocoena spinipinnis*	Rare			1		1		1		3		6	6	0.05	0.0055(0.0172)
*Balaenoptera bonaerensis*	Rare					2		3				5	5	0.04	0.0026(0.0093)
*Stenella coeruleoalba*	Rare	1						4				5	5	0.04	0.0026(0.0080)
*Arctocephalus gazella*	Rare			2		1		1	2			4	4	0.03	0.0061(0.0281)
*Unidentified minke*	Rare							4				4	4	0.03	0.0021(0.0074)
*Ziphius cavirostris*	Rare							3		1		4	4	0.03	0.0019(0.0071)
*Lobodon carcinophaga*	Rare					1		2	3		2	3	3	0.03	0.0017(0.00060)
*Balaenoptera borealis*	Rare							1		2		3	3	0.03	0.0013(0.0045)
*Kogia sima*	Rare			1			1	2				3	3	0.03	0.0040(0.0152)
*Berardius arnuxii*	Rare							1		2		3	3	0.03	0.0019(0.0072)
*Balaenoptera edeni*	Rare							1		1		2	2	0.02	0.0008(0.0036)
*Mesoplodon grayi*	Rare					1		1				2	2	0.02	0.0014(0.0062)
*Mesoplodon densirostris*	Rare	1				1						2	2	0.02	0.0000(0.0000)
*Grampus griseus*	Rare					1		1				2	2	0.02	0.0004(0.0027)
*Balaenoptera physalus*	Rare							1				1	1	0.01	0.0005(0.0030)
*Phocoena dioptrica*	Rare					1						1	1	0.01	0.0008(0.0047)
*Mesoplodon hectori*	Rare					1						1	1	0.01	0.0000(0.0000)
*Mesoplodon layardii*	Rare					1						1	1	0.01	0.0006(0.0036)
*Stenella attenuata*	Rare					1						1	1	0.01	0.0008(0.0050)
*Lagenorhynchus australis*	Rare					1						1	1	0.01	0.0008(0.0052)
*Cephalorhynchus commersonii*	Rare					1						1	1	0.01	0.0008(0.0050)
*Hydrurga leptonyx*	Rare								1			0	0	0.00	-
*Balaenoptera musculus*	Rare						1					0	0	0.00	-
Unknown		7		41	2	274	35	317	66	279	211	918	911	7.75	0.6879(0.6723)
Total		237	4	1238	24	2021	88	5354	369	2943	294	11786	11761	100	8.9171(4.4812)

### Temporal patterns

**Fig 2:**
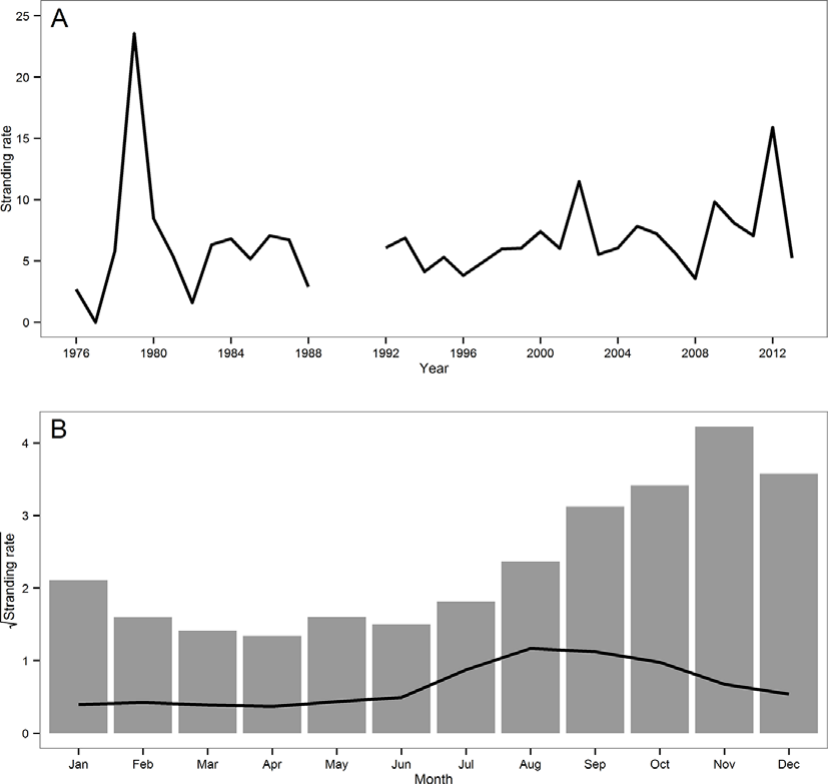
Marine mammal stranding rate by year (A) and month (B) from 1976 to 2013 (records of unidentified species are included). Gray line in (B) represents stranding rate by month after removing records of species with high mortality, migratory whales, neonates of South American fur seal and unidentified marine mammals. Y-axes in (B) are on a square-root scale for ease-of-read purpose. Only stranding records from areas 2 and 3 were included.

Marine mammal stranding rate suggests a perceived increase over 38 year period in the study area (Fig 2A). The overall stranding rate was higher during winter and spring months than during late summer and autumn months (Fig 2B). November and April were the months with the highest (19.8) and lowest (2.6) stranding rate, respectively. After species with known high fishing related mortality (franciscana, bottlenose dolphin and South American sea lion), migratory species (southern right whale, *Eubalaena australis*, humpback whale, *Megaptera novaeangliae*, fin whale, *Balaenoptera physalus*, sei whale, *Balaenoptera borealis*, dwarf minke whale, *Balaenoptera acutorostrata* and Antarctic minke whale, *Balaenoptera bonaerensis*), neonates of South American fur seal and records of unidentified species were removed from the analysis, August (1.3), September (1.2) and October (0.9) had the highest stranding rates while March and April had the lowest rate (0.8) (Fig 2B). Spring had the highest species richness (n = 29 species) followed by autumn (n = 28) and summer and winter (n = 22 each).

Even though Model 1 had the best fit (lowest AIC) for franciscana and South American sea lion, the interaction term was not significant (p = 0.9 and p = 0.1, respectively) (Table 3). For the remaining species, Model 2 had the best fit (Table 3).

**Table 3:** GAM results for each species modeled. Over-dispersion parameter (ϕ).

Species	Model 1			Model 2			Model 3		
	Deviance	ϕ	AIC	Deviance	ϕ	AIC	Deviance	ϕ	AIC
***P*. *blainvillei***	46.3%	0.50	2079.1	43.6%	0.46	2100.0	39.6%	0.41	2122.8
***T*. *truncatus***	16.4%	2.55	720.93	15.8%	2.48	720.00	11%	1.57	732.36
***O*. *flavescens***	9.52%	1.63	1861.26	8.6%	1.59	1866.56	7.57%	1.53	1866.74
***A*. *australis*(neonates)**	62%	1.118	1351.8	62%	1.119	1350.09	57%	0.85	1392.08
***A*. *australis*(juveniles/adults)**	30%	0.86	581.66	30.4%	0.88	577.86	22.7%	0.57	593.11
***A*. *tropicalis***	51.8%	0.38	404.98	51.5%	0.39	402.54	41.2%	0.21	426.37

#### Franciscana

**Fig 3:**
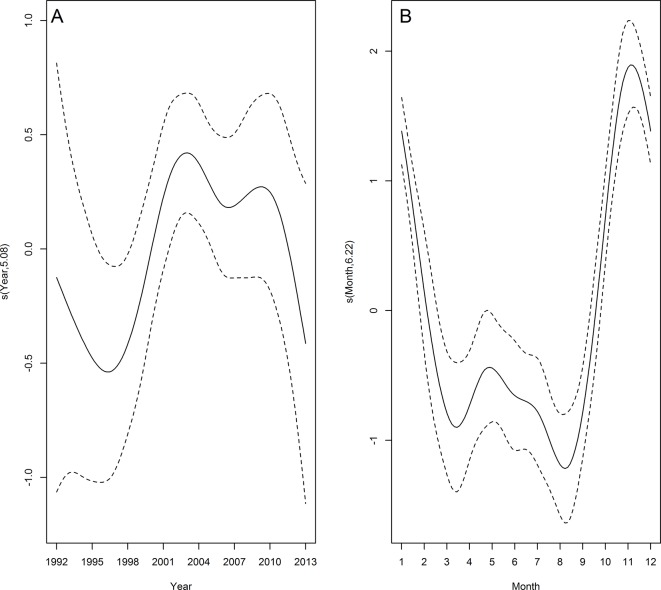
Temporal trends in franciscana strandings. Estimated smooth function (solid line) with 95% confidence interval (dashed lines) for the fitted GAM by year (A) and month (B) from 1992 to 2013. Y-axis = fitted function with estimated degrees of freedom in parentheses.

**Fig 4:**
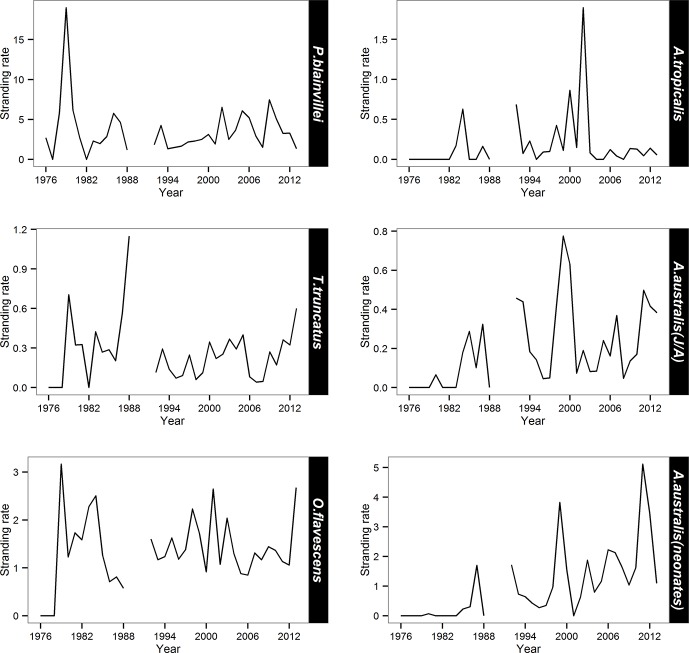
Stranding rate of frequent species by year from 1976 to 2013. Only stranding records from areas 2 and 3 were included.

**Fig 5:**
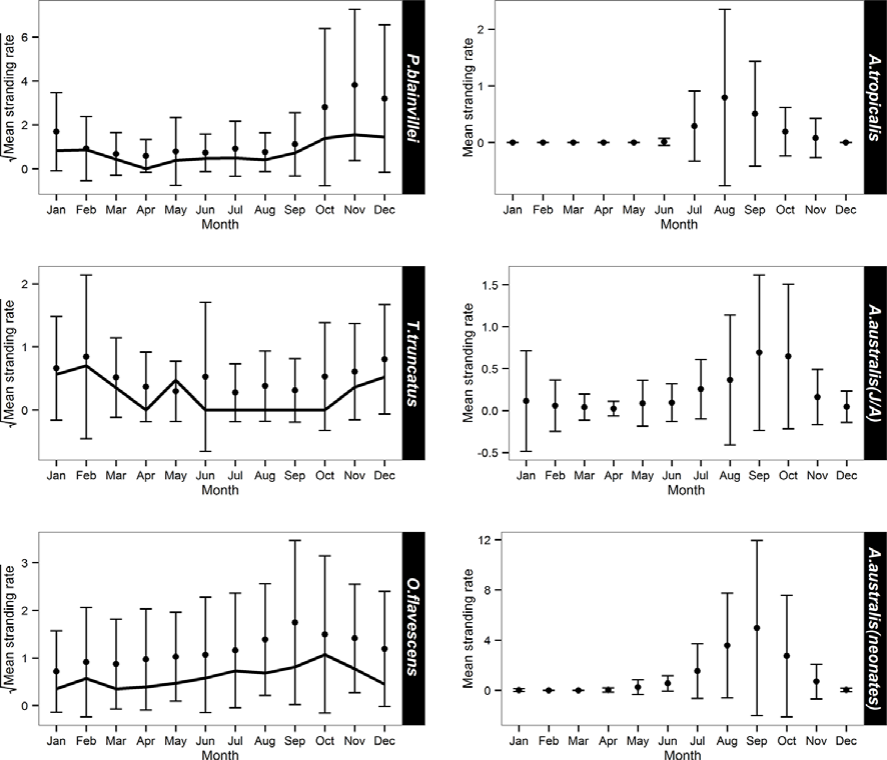
Mean stranding rate of frequent species by month from 1976 to 2013. Black line represents the stranding rate of carcasses in which the cause of death was attributable to bycatch. Y-axes in the left graphics are on a square-root scale for ease-of-read purpose. Only stranding records from areas 2 and 3 were included.

Franciscana had the highest annual mean stranding rate (Table 2). GAM analyses indicated a significantly positive trend in the number of franciscana stranded from 1992 to 2003 and this remained steady until around 2010 when the number began to decline (Figs 3A and 4). Strandings occurred all year round but displayed a conspicuous seasonal pattern with a peak during late spring and early summer (Figs 3B and 5). Temporal predictors accounted for 46.3% of the total variance of franciscana strandings (Table 3). Despite the small proportion of stranding events in which the cause of death was attributable to bycatch (3.1%, n = 133), a similar seasonal pattern was observed when all carcasses of franciscana on the beach were considered (Fig 5).

#### Bottlenose dolphin

**Fig 6:**
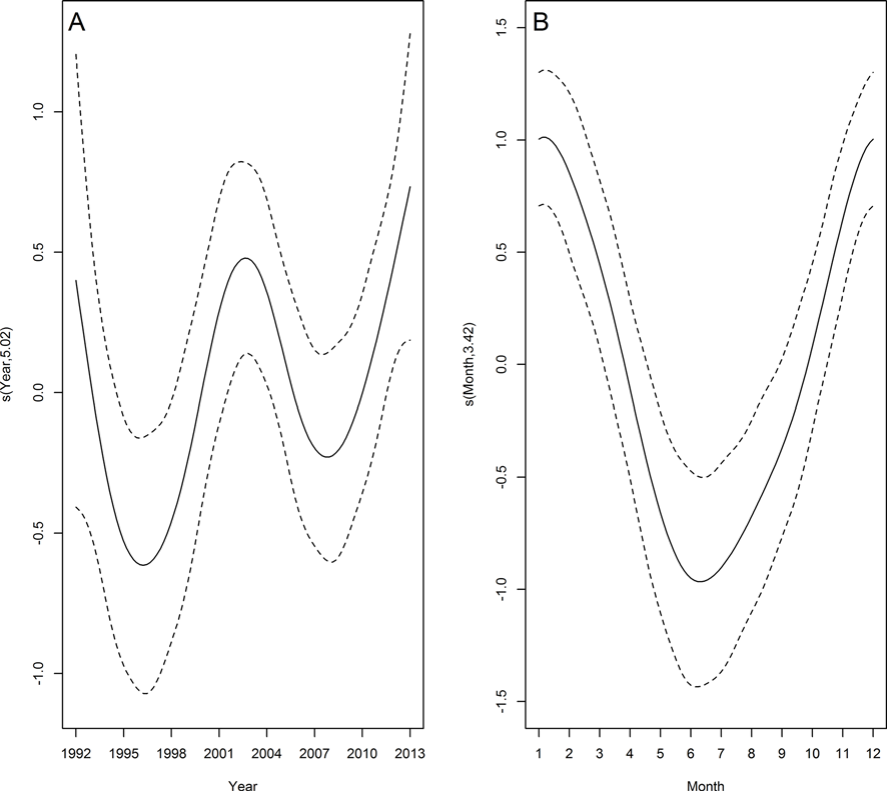
Temporal trends in common bottlenose dolphin strandings. Estimated smooth function (solid line) with 95% confidence interval (dashed lines) for the fitted GAM by year (A) and month (B) from 1992 to 2013. Y-axis = fitted function with estimated degrees of freedom in parentheses.

The stranding rate of bottlenose dolphin during the first 13 years showed three peaks: 1979, 1983 and 1988 (Fig 4). The fitted model adjusted for the period between 1992 and 2013 indicated an increase in strandings between 1996 and 2003, then a decrease till 2008 (Fig 6A). The number of stranding records was highest during spring and summer and lowest during autumn and winter (Figs 5 and 6B). The temporal predictors accounted for 15.8% of the total variance of bottlenose dolphin strandings (Table 3). Despite the small proportion of stranding events in which the cause of death was attributable to bycatch (8.7%; n = 22), a similar seasonal pattern was observed when all carcasses of bottlenose dolphin on the beach were considered (Fig 5).

#### South American sea lion

**Fig 7:**
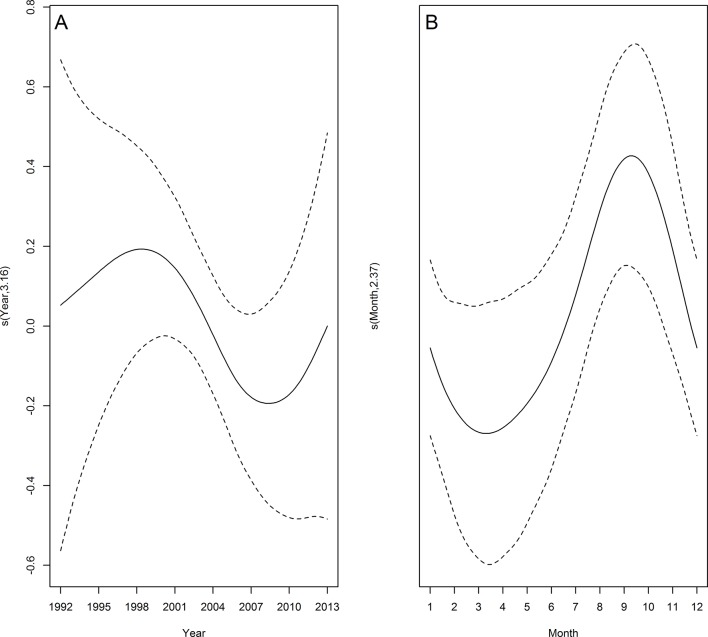
Temporal trends in South American sea lion strandings. Estimated smooth function (solid line) with 95% confidence interval (dashed lines) for the fitted GAM by year (A) and month (B) from 1992 to 2013. Y-axis = fitted function with estimated degrees of freedom in parentheses.

The inter-annual stranding rates for South American sea lion from 1979 to 1988, showed greater variation compared to the period from 1992 to 2013 (Fig 4). Despite the improvement of the model fit (Model 1) year was non-significant (p = 0.17) (Fig 7A). The fitted GAM adjusted for the period between 1992 and 2013 showed the highest number of strandings in winter and spring (Figs 5 and 7B). The temporal predictors accounted for 9.52% of the total variance of South American sea lion strandings. As observed for the two previous species, a similar seasonal pattern was observed between records with signs of fishery interaction (3.4%; n = 67) and when all carcasses were considered (Fig 5).

#### South American fur seal

**Fig 8:**
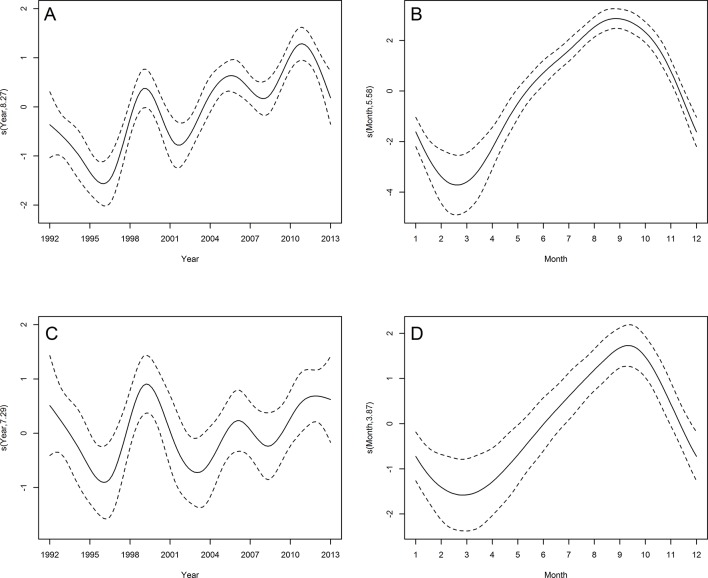
Temporal trends in South American fur seal strandings. Estimated smooth function (solid line) with 95% confidence interval (dashed lines) for the fitted GAM by year and month from 1992 to 2013. Neonates (A and B) and juveniles/adults (C and D). Y-axis = fitted function with estimated degrees of freedom in parentheses.

Among pinniped species, South American fur seal displayed the highest mean stranding rate (Table 2). GAM analyses adjusted for the period between 1992 and 2013 showed a temporal increase in the number of South American fur seal strandings for both categories (adults/juveniles and neonates) (Fig 8A and 8C). The three peaks observed in the inter-annual trend were identical for both categories (Fig 8A and 8C). Considering seasonality, the peak of strandings occurred in September for both categories (Figs 5 and 8B and 8D). The temporal predictors accounted for 30.4% (juveniles/adults) and 62% (neonates) of the total variance of South American fur seal strandings (Table 3).

#### Subantarctic fur seal

**Fig 9:**
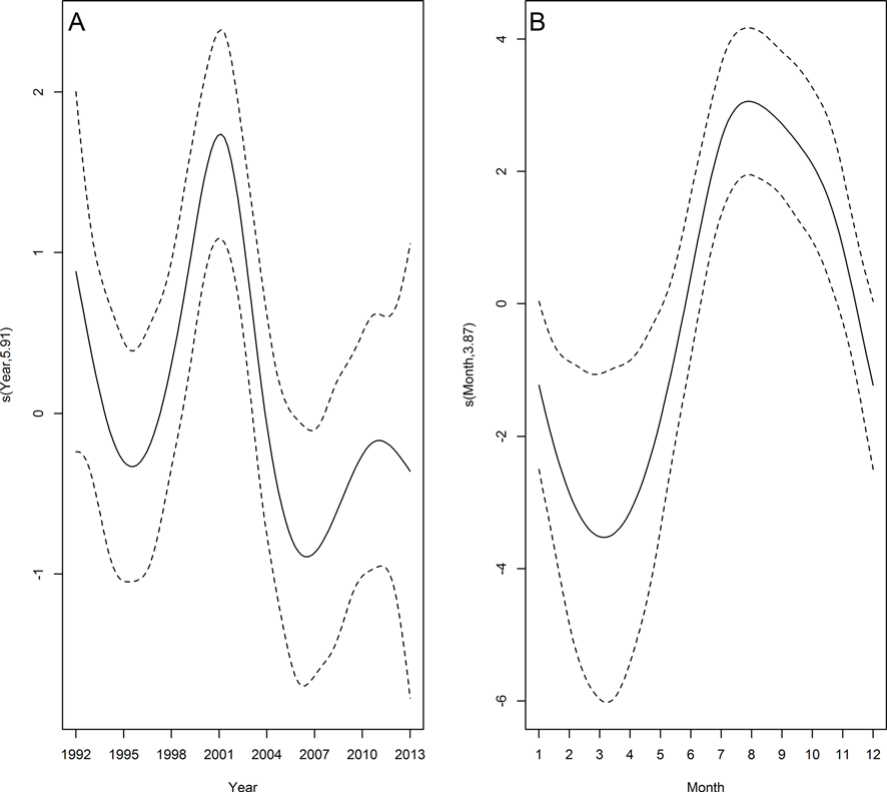
Temporal trends in subantarctic fur seal strandings. Estimated smooth function (solid line) with 95% confidence interval (dashed lines) for the fitted GAM by year (A) and month (B) from 1992 to 2013. Y-axis = fitted function with estimated degrees of freedom in parentheses.

From 1976 to 1982 no stranding of subantarctic fur seal was recorded (Fig 4). According to the fitted GAM adjusted for the period between 1992 and 2013, the stranding showed a slight decline except for a peak in 2001 (Fig 9A). The GAM model indicated a strong seasonal stranding pattern with higher values occurring during winter, especially August (Figs 5 and 9B). No records were observed in summer and early autumn (Fig 5). The temporal predictors accounted for 51.5% of the total variance of subantarctic fur seal strandings (Table 3).

#### Other species

**Fig 10:**
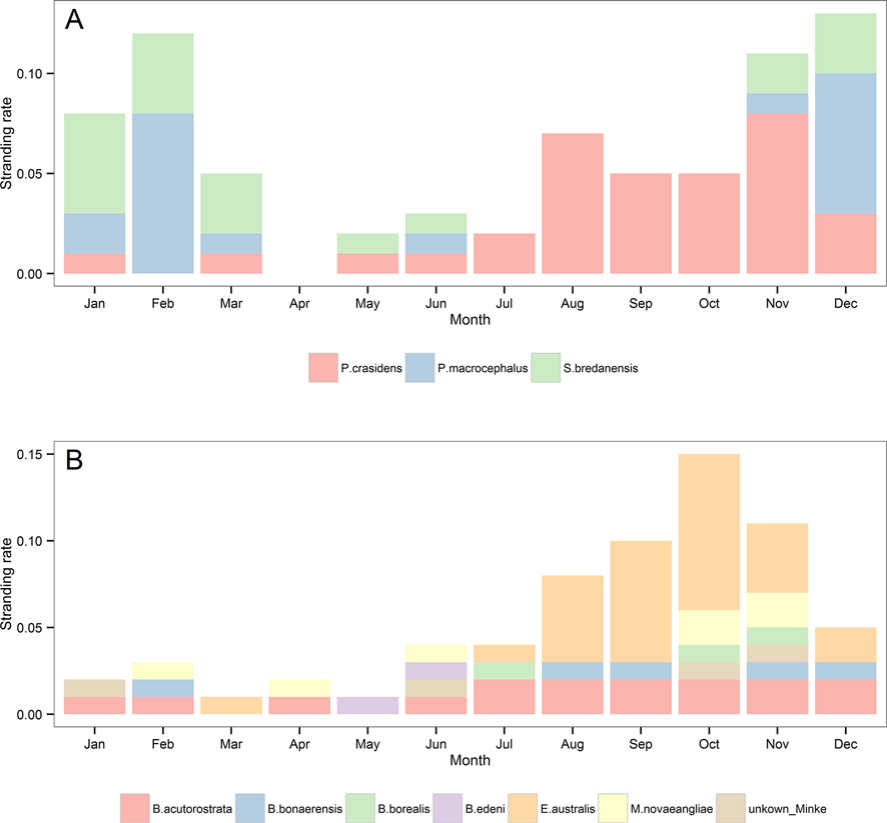
Stranding rates of false killer whale, sperm whale and rough-toothed (A) and baleen whales (B) by month from 1976 to 2013. Stranding records from all areas (1–5) were included.

The occasional odontocete species (Table 2) did not show any clear annual and or seasonal trend. However, false killer whale, *Pseudorca crassidens*, had a higher stranding rate during winter and spring, sperm whale, *Physeter macrocephalus*, from late spring to mid-summer and rough-toothed dolphin, *Steno bredanensis*, in summer (Fig 10).

Among baleen whales (Mysticeti), Southern right whale stranded most often, followed by dwarf minke, humpback, Antarctic minke, sei, Bryde’s, *Balaenoptera edeni*, and blue, *Balaenoptera musculus*, and fin whales (Table 2). Most stranding events of baleen whales were recorded after 1990 (Table 2) and were more frequent in September, October and November (Fig 10B).

**Fig 11:**
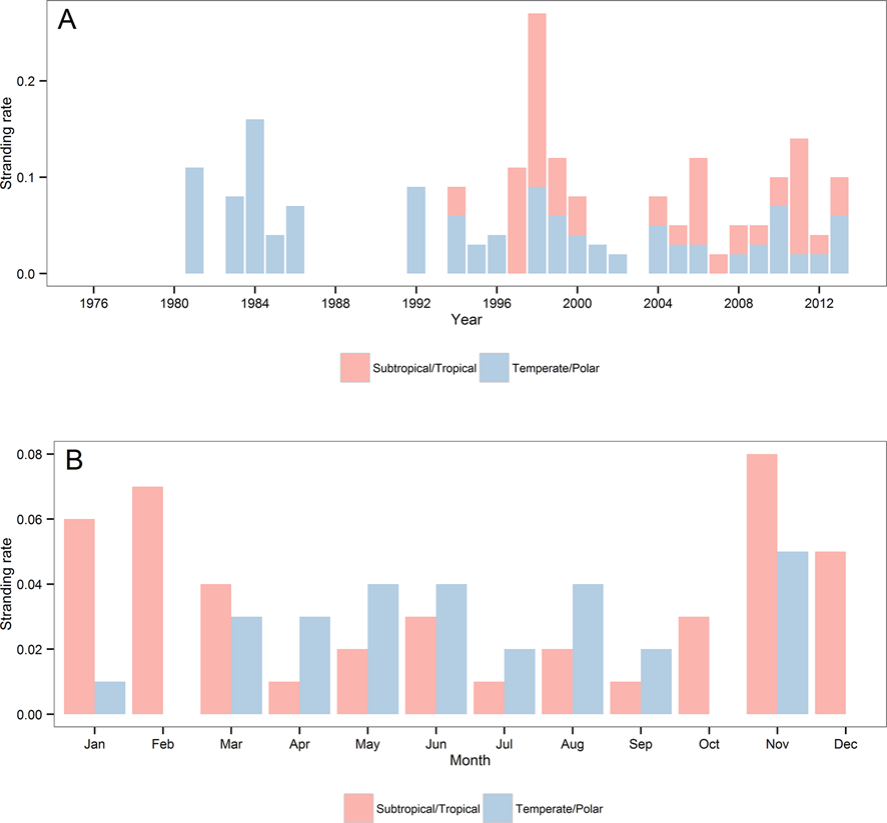
Stranding rates of tropical/subtropical and temperate/polar species by year (A) and month (B) from 1976 to 2013. Tropical/subtropical species: pantropical spotted dolphin, *Stenella attenuata*, Atlantic spotted dolphin, *Stenella frontalis*, rough-toothed dolphin, Fraser’s dolphin, *Lagenodelphis hosei* and *Bryde’s whale*. Temperate/polar species: Burmeister’s porpoise, *Phocoena spinipinnis*, spectacled porpoise, *Phocoena dioptrica*, Peale’s dolphin, *Lagenorhynchus australis*, Commerson’s dolphin, *Cephalorhynchus commersonii*, southern elephant seal, *Mironga leonina*, crabeater seal, *Lobodon carcinophaga*, Antarctic fur seal, *Arctocephalus gazella*, Gray’s Beaked whale *Mesoplodon grayi*, strap-toothed whale, *Mesoplodon Layardii*, and Arnoux’s beaked whale, *Berardius arnuxii*. Stranding records from all areas (1–5) were included.

The remaining rare species were either typically subtropical/tropical, temperate/polar or wide-ranging, deep-water cetaceans (Table 2). Temperate/polar species were recorded since the early 1980s, while for tropical/subtropical species only after 1993 (Fig 11A). For both Temperate/polar and subtropical/tropical species no clear inter-annual trend were observed (Fig 11A). As expected, the seasonal stranding rates for both subtropical/tropical and temperate/polar species showed an opposite pattern, with the former stranding mostly during spring and summer and the latter during autumn and winter (Fig 11B).

## Discussion

Long-term systematic beach surveys shed light on many aspects of marine mammal stranding patterns in southern Brazil. Although caution is needed when interpreting stranding data [6,42,43], the results of this study are likely to reflect the broad pattern of marine mammal occurrence in this sector of the subtropical western South Atlantic. The diverse array of stranded species suggests a high richness of marine mammals in this region. Nevertheless, despite the richness (n = 40), only five species were involved in 97% of the total strandings. This high proportion is presumably related to the coastal habitat of those species and, for some of them, the high incidence is due to fishing-related mortality [6]. The southern Brazilian continental shelf is flat, with width varying from 100 to 180km. Therefore, typically offshore species inhabiting the outer shelf and beyond are less likely to wash ashore. The occurrence of deep-water species in the stranding records was very low, probably because they are more likely to decompose and sink before they reach the shore (*e*.*g*. [43]).

Marine mammal distribution is to a great extent related to prey distribution [1]. In general, areas of high productivity appear to attract top predators (*e*.*g*. [54,55]). Therefore, seasonal changes in prey availability can cause changes in marine mammal occurrence. After removing species with known high fishing-related mortality, migratory species and neonates of South American fur seal from the analyses, late winter and early spring were the seasons with the highest stranding rates. This pattern could be associated with an increase in biological productivity during these seasons. Phytoplankton biomass over the Brazilian continental shelf is higher in winter and spring and has been shown to be related to nutrient supply from Subantarctic Water carried by the MFC and from freshwater discharge of the La Plata River [23,25]. The high primary productivity during winter and spring supports a high biomass of demersal and pelagic fish (*e*.*g*. *Cynoscion guatucupa*, *Trichiurus lepturus*, *Engraulis anchoita*) and squid (*e*.*g*. *Loligo sanpaulensis*, *Illex argentinus*) that migrate from the south in association with the Subtropical Convergence [56,57]. These species are important prey for many marine mammals (*e*.*g*. [58–60]), thus an increase in abundance and diversity of predator species is to be expected during these seasons. Shipboard surveys for marine mammals were carried out in autumn and spring (2009–2014) on the southern Brazilian outer continental shelf and slope and higher abundance and species richness were observed in spring (ECOMEGA unpubl. data), which is consistent with the stranding pattern observed in this study.

### Frequent species

Some of the five species that frequently stranded in this region (franciscana, bottlenose dolphin, South American and subantarctic fur seals and South American sea lion) are either primarily coastal or highly vulnerable to fishery-related mortality. Franciscana and bottlenose dolphins are frequently killed in commercial and artisanal gillnetting, respectively [6,20,35]. The peak of franciscana strandings during late spring and early summer coincides with high gillnet fishing effort targeting the white croaker (*Micropogonias furnieri*) near shore [35,37,39], where franciscanas are most abundant [61,62]. The inflated stranding rate observed in 1979 is probably because beach surveys were only conducted in months of high bycatch (*e*.*g*. [6,19,39]). Following the collapse of some fish stocks by the late 1980s and early 1990s fishermen increased substantially gillnet lengths to compensate for lower catches per unit of effort (*e*.*g*. [27,37,39]). As a consequence franciscana bycatch and strandings have also increased until 2003 (see Fig 3). Although artisanal gillnetting occurs year-round inside the Patos Lagoon Estuary and along the adjacent marine coast, the overlap between gillnets and dolphins is higher in spring and summer [63], suggesting that the highly seasonal stranding pattern of bottlenose dolphin is related to bycatch in fisheries [20]. This might explain the very high stranding rate observed in 1988 that, similarly to what happened to franciscana in 1979, is likely due to low beach survey effort conducted in months of higher bycatch [20]. The inter-annual variability in strandings may be also related to the pattern of fishing effort [20]. Current levels of fishing-related mortality of these species may not be sustainable [20,36,38,64]. In 2012, the Brazilian Government published a norm to regulate gillnet fisheries, reduce fishing effort and establish some no-fishing zones aimed at protecting certain vulnerable species, including coastal cetaceans. Therefore a decrease in stranding rate of both franciscanas and bottlenose dolphins after the end of 2013, when this norm was implemented, is expected. It is important to emphasize, however, that the decrease in franciscana stranding after 2010, may be associated with population decline and carefully interpretation of stranding trends is needed.

Three otariid seals are often recorded along the Brazilian coast: South American sea lion, South American and subantarctic fur seals. The first two are the most widely distributed otariids in the Southern Hemisphere, with breeding colonies extending from Peru to Uruguay [18,65], while subantarctic fur seal breeds on islands north of the Antarctic Convergence [66]. Specimens of South American fur seal and the majority of vagrant individuals of subantarctic fur seal found on the Brazilian coast are from Uruguay and Gough Island populations, respectively [66, 67]. Based on what is known about the biology of South American sea lion, it is presumed that individuals in Brazilian waters come from the Uruguayan breeding colonies [68]. The higher frequency of occurrence of the two fur seals species on the southern Brazilian coast during winter and early spring is probably related to post-reproductive dispersal, with the benefit of the northward flow of the MFC and displacement of the Subtropical shelf front in winter [22,24,69]. All sightings of subantarctic fur seal in the Indian Ocean occurred in winter and the northward flow of the South Indian Ocean current is considered one of the main factors to explain the presence of this species in tropical and subtropical regions of that ocean [70–72]. Since many sub-adult and adult individuals of South American sea lion concentrate throughout the year in non-breeding rookeries in southern Brazil [73,74], the seasonality of its occurrence is less pronounced than that of the fur seal species.

Although South American sea lion also breed on the Uruguayan coast, only neonates of South American fur seal are found stranded in large numbers on the southern Brazilian coast. The high stranding rates of neonates occur in August and September and coincide with the immediately post-weaning period.

An estimated annual population growth rate of 2% since 1991, when harvesting of South American fur seal stopped on the Uruguayan coast [75], may explain the increased number of strandings in recent years. Despite the general increase in strandings, however, fluctuations were observed. High stranding rates of neonates in 1999 and 2003 were preceded by years with high numbers of births (65,000 and 72,000) and low stranding rates in 1992 and 1993 followed years with lower reported numbers of births (48,000 and 50,000) [76]. However, the low and moderate stranding rates in 1996 and 2005, respectively, were also preceded by years with high numbers of births (68,000 and 93,000) [76]. Determining which factors influence post-weaning survival is often difficult, but they are probably related to the environmental conditions that influence food supply. For example, the highest stranding rates were observed in 1999 and 2011 when moderate and strong La Niña events occurred. During La Niña, productivity on the southern Brazilian continental shelf decreases [23] and lower food availability is expected.

Although the exploitation of South American sea lion in Uruguay ceased in 1978, the population seems to be decreasing [77]. Fishing-related mortality has been identified as the major cause of this decline [77,78]. As observed for franciscana and bottlenose dolphin, the similar pattern between records with signs of fishing-related mortality and all records (with and without evidence of fishery interactions) suggest that fishery is the major source of South American sea lion mortality in southern Brazil. The higher stranding rates observed in late winter and early spring may be related to the increase in trawl and gillnet fishing effort during this period which coincides with post-reproductive dispersal of this species. The overlap of foraging habitat of sea lions with fishing grounds increases the risk of both bycatch and intentional killing. Fishermen often shoot sea lions that remove fish from their nets in this area [73]. Similar to franciscana, the very high stranding rate observed in 1979 is probably because beach surveys were only conducted in months of high bycatch (*e*.*g*. [21]).

Anomalies in ocean currents and other phenomena (*e*.*g*. ENSO) have been suggested as possible explanations for the occurrence of subantarctic fur seal on the Brazilian coast [79,80]. Short- to medium-term fluctuations in climate due to ENSO events can produce anomalous environmental conditions that drastically change marine productivity and, consequently, affect the foraging patterns of top predators [81,82]. In the Southern Ocean some studies have shown that the warm phase of ENSO negatively affects the recruitment and biomass of krill and fish, in some cases forcing top predators to move far from their breeding area in search of food (*e*.*g*. [83,84]). However, the low stranding rates of subantarctic fur seal observed during the strongest El Niño episodes of 1982/1983, 1997/1998 and 2009/2010 and the high stranding rates that coincided with a strong La Niña event in 2000 and a moderate El Niño event in 2002 suggest that the main factors influencing inter-annual variability of subantarctic fur seal occurrence in Brazilian coastal waters are still unknown. The increased occurrence of these fur seals on and around South American, African and Indian Ocean islands, for example, has been attributed to population expansion (*e*.*g*. [70–72,85]). However, the slight decrease in stranding along the years may be associated with climate change (see discussion regarding subtropical/tropical and temperate/polar species).

### Other species

Strandings of occasional species consisted, to a great extent, of cetaceans that typically inhabit offshore waters. Therefore, the relatively low stranding rates do not necessary mean that the abundance of those species is low. The very wide continental of southern Brazil reduces the chances of offshore species washing ashore. Information based on opportunistic and systematic at-sea surveys for marine mammals as well as on records of incidental catches in fishing gear have shown that sperm whale, short-beaked common dolphin, *Delphinus delphis*, long-finned pilot whale, *Globicephala melas*, false killer whale and killer whale, *Orcinus orca* are common over the southern Brazilian outer continental shelf and slope [31,86–90] (ECOMEGA unpubl. data). The low stranding rates of some species, however, does reflect their low abundance in the subtropical western South Atlantic (*e*.*g*. Fraser’s and Atlantic spotted dolphins).

For false killer whale the higher stranding rate in winter/spring might be related to the higher productivity, which could attract the animals to this region. Analysis of killer and false killer whales depredation on the Uruguayan pelagic longline fishery operating in the Southwestern Atlantic Ocean near the study area revealed the most interaction occur during winter and spring [91]. Stomach content and stable isotopes studies suggest that at least some false killer whale in southern Brazil use coastal waters, mainly in spring, to feed upon abundant sciaenid fish [17,92]. During this period the fishing effort over the southern Brazilian continental shelf increases [27] and therefore an increase in marine mammal bycatch is expected. Year-round occurrence of sperm whale over the continental slope off southern Brazil suggests that this region is an important feeding ground for sperm whales [31,90] (ECOMEGA unpubl. data). Nevertheless, the reasons for the higher stranding rate during summer remain to be elucidated. The higher stranding rate of rough-toothed dolphin during summer might be related to the major influence of tropical water on the southern Brazilian continental shelf [93].

The reasons for the increased stranding rates of baleen whales, especially after the 1990s, are unclear but might reflect the recovery of some population after the International Whaling Commission’s moratorium on commercial whaling came into effect. For example, both Southern right and humpback whales are showing high estimated annual growth rates in the western South Atlantic (*e*.*g*. [94,95]). The higher stranding rate of southern Right whale compared with other baleen whales is likely due to its coastal migration route to and from its wintering grounds in southern Brazil. During winter and early spring southern right whales use shallow waters and protected bays along southern Brazil for breeding and nursing the calves [96,97]. Stranding records of southern Right whale in this area, between 1977 and 1995, were most frequent in October followed by August and September [98]. Despite its coastal distribution, the stranding rate of humpback whale was low in southern Brazil because its migration route between breeding (northeastern Brazil) and foraging areas (South Georgia/South Sandwich Island) is far offshore in southern Brazil [99,100].

Although migratory pattern of blue, fin, sei and minke whales in Southern Atlantic Ocean is not well documented a review of the occurrence and distribution of the genus *Balaenoptera* along the Brazilian coast revealed that most whales are observed during the austral winter and spring, indicating a seasonal pattern of occurrence of this genus for Southwestern Atlantic Ocean [101]. Stranding records outside the breeding season may reflect differences in the timing of migration within and between species or the residency of some individuals in tropical and subtropical areas throughout the year. Records of juvenile dwarf minke whale throughout much of the year indicate that some individuals do not migrate to Antarctic or sub-Antarctic waters [101] and may exploit coastal areas off eastern South America with locally high productivity (*e*.*g*. [102]). Balaenopterids are known to feed outside their regular high-latitude feeding grounds when plenty of prey is available in other areas [103]. Bryde’s whale does not migrate to polar/subpolar feeding grounds [104] and is relatively common in the coastal upwelling ecosystem off south-eastern Brazil [101,105], with occasional occurrence in the study area [18] (ECOMEGA, unpubl. data).

The remaining rare species were either typically subtropical/tropical, temperate/polar species or wide-ranging deep-water cetaceans. Therefore, for some widely distributed species such as *Kogia* spp., Risso’s dolphin, *Grampus griseus*, and Cuvier’s Beaked whale, *Ziphius cavirostris*, the low stranding rate may be explained by either their offshore distribution or their low regional abundance [18,106,107]. Despite the temperate/polar marine mammals have not shown a clear temporal pattern, the lack of records of subtropical/tropical species until approximately the mid 1990s followed by their relatively frequent occurrence after this period might be at least partly associated with climate change. From 1993 to 2002 the spatial distribution of SST anomalies reveals that warming occurred across the South Atlantic basin between 24°S and 40°S [108]. According to the same authors, from October 1992 to December 2007 a southward shift of 0.6 to 0.9° decade ^-1^ was found in the latitude of the BC/MFC confluence.

Despite the difficulties of interpreting stranding data given that stranded carcasses can be found on the beach as a result of many processes (*e*.*g*. at-sea mortality, buoyancy, drift, and detection probability) [6,42–45], long time series derived from consistent beach survey effort can contribute to monitoring of marine mammals. Stranding data can document species occurrence and reveal changes in mortality rates or shifts in distribution due to oceanic conditions. Although in the present study we did not quantify the relationship between fishing effort and stranding data, the seasonal coincidence between high stranding rates of some species (*e*.*g*. bottlenose dolphin, South American sea lion and franciscana) and high fishing effort suggests a plausible link. Establishing a definite causal link between climate change and species/community ecology is difficult. Despite the time series used here is still short in duration (in relation to non-stationary long-term duration change signals) to allow for confident predictions about the possible ecosystem consequences of long term climate change, our interest here was to provide evidence that short-term sub-decadal signals in climate variability may be affecting the patterns of marine mammal occurrence in the Southwester Atlantic Ocean. It is important to emphasize that biological response to short and medium term signals in climate variability may be the best opportunity to explore how biological communities respond to changes [80]. Continued beach surveys are essential to evaluate trends in fishing-related mortality and to further explore the relationships between species distribution patterns and oceanic processes at different time-scales in the western South Atlantic.

## Supporting Information

S1 Fig Number of times that beach surveys were carried out (including partial surveys) in each area from 1976 to 2013. I = 84km; II = 51km; III = 63km; IV = 70km; V = 87km.
(TIFF)

S1 Table Marine mammal mass strandings (*n* = 7) during 1976–2013 in southern Brazil. (DOCX)

## References

[1] Forcada J. Distribution In: Perrin WF, Wursig B and Thewissen TGM, editors. Encyclopedia of marine mammals. San Diego: Academic Press; 2009 p. 316–321.

[2] MacLeod CD, Pierce GJ, Santos MB. Geographic and temporal variations in strandings of beaked whales (Ziphiidae) on the coasts of the UK and the Republic of Ireland from 1800–2002. J Cetacean Res Manag. 2004; 6(1): 79–86.

[3] Pyenson ND. Carcasses on the coast: measuring the ecological fidelity of the cetacean stranding record in eastern North Pacific Ocean. Paleobiology. 2010; 36: 453–480.

[4] Maldini D, Mazzuca L, Atkinson S. Odontocete stranding patterns in the main Hawaiian Islands (1937–2002): how do they compare with live animal surveys? Pac Sci. 2005; 59(1): 55–67.

[5] Leeney RH, Amies R, Broderick AC, Witt MJ, Loveridge J, Doyle J, et al Spatio-temporal analysis of cetacean strandings and bycatch in a UK fisheries hotspot. Biodivers Conserv. 2008; 17: 2323–2338.

[6] Prado J, Secchi ER, Kinas PG. Mark-recapture of the endangered franciscana dolphin (*Pontoporia blainvillei*) killed in gillnet fisheries to estimate past bycatch from time series of stranded carcasses in southern Brazil. Ecol Indic. 2013; 32: 35–41.

[7] Harwood J. What killed the Mediterranean monk seals? Nature. 1998; 393: 17–18. 9590683 10.1038/29877

[8] Fire SE, Wang Z, Byrd M, Whitehead HR, Paternoster J, Morton SL. Co-occurrence of multiple classes of harmful algal toxins in bottlenose dolphins (*Tursiops truncatus*) stranding during an unusual mortality event in Texas, USA. Harmful Algae. 2011; 10(3): 330–336.

[9] Castello HP, Piñero ME. Variamentos de cachalote, *Physeter catodon*, en las costas del Atlantico Del Brasil y La Argentina (Cetacea, Physeteridae). Physis Secc. A. Buenos Aires. 1974; 86: 371–374.

[10] Gianuca NM, Castello HP. First record of the southern bottlenose whale, *Hyperodon planifrons* from Brazil. Sci Rep Whales Res Inst. 1976; 28: 119–126.

[11] Castello HP, Pinedo CP. Os visitantes ocasionais de nosso litoral. Natureza em Revista. 1977; 3: 40–46.

[12] Castello HP, Pinedo MC. *Mesoplodon densirostris* (Cetacea, Ziphidae), primeiro registro para o Atlântico Sul Ocidental. Bolm Inst Oceanogr. 1980; 29(2): 91–94.

[13] Pinedo MC. A note on a stranding of the humpback whale on the southern coast of Brasil. Sci Rep Whale Res Inst. 1985; 36: 165–168.

[14] Pinedo MC. First record of a dwarf sperm whale from southwest Atlantic, with reference to osteology, food habits and reproduction. Sci Rep Whale Res Inst. 1987; 38: 171–186.

[15] Pinedo MC. Primeiro registro de *Phocoena spinipinnis* (Cetacea, Phocoenidae) para o litoral do Rio Grande do Sul, Brasil, com medidas osteológicas e análise do conteúdo estomacal. Atlântica. 1989; 11(1): 85–99.

[16] Rosa FCW, Pinedo MC. Nota sobre a ocorrência do cachalote pigmeu, *Kogia breviceps*, no litoral do Rio Grande do Sul, Brasil. Atlântica. 1989; 11(1) 109–113.

[17] Pinedo MC, Rosa FCW. Novas ocorrências de *Pseudorca crassidens* (Cetacea, Delphinidae) para o Atlântico Sul Ocidental com observação sobre medidas cranianas e alimentação. Atlântica. 1989; 11(1): 77–83.

[18] Bastida R, Rodríguez D, Secchi ER, Silva V. Mamiferos acuaticos -Sudamerica Antartida. 1st ed. Buenos Aires: Vazquez Mazzini; 2007.

[19] Pinedo MC, Polacheck P. Trends in franciscana (*Pontoporia blainvillei*) stranding rates in Rio Grande do Sul, southern Brazil (1979–1998). J Cetacean Res Manag. 1999; 1(2): 179–189.

[20] Fruet PF, Kinas PG, Silva KG, Di Tullio JC, Monteiro DS, Rosa LD, et al Temporal trends in mortality and effects of by-catch on common bottlenose dolphins, *Tursiops truncatus*, in southern Brazil. J Mar Biol Assoc UK. 2012; 92: 1865–1876.

[21] Kinas PG, Silva KG, Estima SC, Monteiro DS. Generalized linear models applied to stranding data of South American Sea Lions (*Otaria flavescens*) and South American Fur Seals (*Arctocephalus australis*) in Southern Brazil. Lat Am J Aquat Mamm. 2005; 4(1): 7–14.

[22] Lima ID, Garcia CA, Möller OO. Ocean surface processes on the southern Brazilian shelf: characterization and seasonal variability. Cont Shelf Res. 1996; 16(10): 1307–1317.

[23] Ciotti AM, Odebrecht C, Fillmann G, Möller OO. Freshwater outflow and Subtropical Convergence influence on phytoplankton biomass on the southern Brazilian continental shelf. Cont Shelf Res. 1995; 15(14): 1737–1756.

[24] Möller OO, Piola RA, Freitas AC, Campos EJD. The effects of river discharge and seasonal winds on the shelf off southeastern South America. Cont Shelf Res. 2008; 28: 1607–1624.

[25] Muelbert JH, Acha M, Mianzan H, Guerrero R, Reta R, Braga ES, et al Biological, physical and chemical properties at the Subtropical Shelf Front Zone in the SW Atlantic Continental Shelf. Cont Shelf Res. 2008; 28: 1662–1673.

[26] Castello JP, Duarte AK, Möller OO, Niencheski F, Odebrecht C, Weiss G, et al 1990. On the importance of coastal and subantarctic waters for the shelf ecosystem off Rio Grande do Sul. São Paulo: Proceedings of ACIESP. 1990; 1(1): 112–129.

[27] Haimovici M, Castello JP, and Vooren CM. Pescarias In Seeliger U, Odebrecht C, Castello JP, editors. Os ecosistemas costeiro e marinho do extremo sul do Brasil. Rio Grande: Ecoscientia; 1998 p. 205–218.

[28] Petry MV, Fonseca VSS. Effects of human activities in the marine environmental on seabirds along the coast of Rio Grande do Sul, Brazil. Ornitol. Neotrop. 2002; 13: 137–142.

[29] Bugoni L, Mancini PL, Monteiro DS, Nascimento L, Neves TS. Seabird bycatch in the Brazilian pelagic longline fishery and a review of capture rates in the Southwestern Atlantic Ocean. Endanger. Species Res. 2008; 5: 137–147.

[30] Zerbini NA, Secchi ER, Bassoi M, Dalla Rosa L, Higa A, Souza L, et al Distribuição e abundância relativa de cetáceos na zona econômica exclusiva da região Sudeste-Sul do Brasil São Pulo: Instituto–USP (Série documentos Revizee: Score Sul. 2004.

[31] Pinedo MC, Polacheck T, Barreto AS, Lammardo MP. A note on vessel of opportunity sighting surveys for cetaceans in the shelf edge region off the southern coast of Brazil. J Cetacean Res Manag. 2002; 4(3): 323–329.

[32] Vooren CM. Elasmobrânquios Demersais In Seeliger U, Odebrecht C, Castello JP, editors. Os ecosistemas costeiro e marinho do extremo sul do Brasil. Rio Grande: Ecoscientia; 1998 p.157–162.

[33] Hall MA, Alverson DL, Metuzals KL. By-Catch: Problems and Solutions. Mar Pollut Bull. 2000; 41(1): 204–219.

[34] Reeves RR, McClellan K, Werner TB. Marine mammal bycatch in gillnet and other entangling net fisheries 1990 to 2011. End. Spe. Res. 2013; 20(1): 71–97.

[35] Secchi E R, Kinas PG, Muelbert M. Incidental catches of franciscana in coastal gillnet fisheries in the franciscana management area III: period 1999–2000. Lat Am J Aquat Mamm. 2004; 3(1): 61–68.

[36] Secchi ER, Fletcher D. Modelling population growth and viability analysis for four franciscana stocks: effects of stock-specific differences in life traits, fishing bycatch, parameter uncertainty and stochasticity. Cambridge. International Whaling Commission, Scientific Committee Paper SC/56/SM20. 2004.

[37] Secchi ER, Zerbini AN, Bassoi M, Dalla Rosa L, Möller LM, Campos CCR. Mortality of franciscanas, *Pontoporia blainvillei*, in coastal gillneting in southern Brazil: 1994–1995. Rep Int Whal Commn. 1997; 47: 653–658.

[38] Kinas PG. The impact of incidental kills by gillnets on the franciscana dolphin (*Pontoporia blainvillei*) in southern Brazil. B Mar Sci. 2002; 70: 409–421.

[39] Ferreira EC, Muelbert MMC, Secchi ER. Distribuição espacial-temporal das capturas acidentais de toninhas (*Pontoporia blainvillei*) em redes de emalhe e dos encalhes ao longo da costa sul do Rio Grande do Sul, Brasil. Atlântica. 2010; 32: 183–197.

[40] Szephegyi MN, Franco-Trecu V, Doño F, Reyes F, Forselledo R, Crespo EA. 2010. Primer relevamento sistemático de captura incidental de mamíferos marinos en la flota de arrastre de fondo costero de Uruguay: XIV Reunión de Trabajo de Expertos en Mamíferos Acuáticos de América Del Sur y 8° Congreso de la Sociedad Latino Americana de Especialistas en Mamíferos Acuáticos (SOLAMAC), Florianópolis, Abstract. 2010.

[41] CEPERG. Desembarque de pesca no Rio Grande do Sul de 1997 a 2011: Available: http://www.icmbio.gov.br/ceperg/images/stories/publicacoes/titulo2.pdf.

[42] Authier M, Peltier H, Dorémus, Dabin W, Canneyt OV, Ridoux V. How much are stranding records affected by variation in reporting rate? Case study of small delphinids in Bay of Biscay. Biodivers Conserv. 2014; 23: 2591–2612.

[43] Peltier H, Dabin W, Daniel P, Canneyt OV, Dorémus G, Huon M, et al The significance of stranding data as indicators of cetacean population at sea: Modelling the drift of cetacean carcasses. Ecol. Indic. 2012; 18: 278–290.

[44] Epperly Sp, Braun J, Chester AJ, Cross FA, Merriner JV, Teste P, et al Beach stranding as an indicator of at-sea mortality of sea turtles. Bull. Mar. Sci. 1996; 59: 289–297.

[45] Hart KM, Mooreside P, Crowder LB. Interpreting the spatio-temporal patterns of sea turtle stranding: Going with flow. Bio Conserv. 2006; 129: 283–290.

[46] Calliari L.J. 1998. Ambientes Costeiros e Marinhos e sua Biota In Seeliger U, Odebrecht C, Castello JP, editors. Os ecosistemas costeiro e marinho do extremo sul do Brasil. Rio Grande: Ecoscientia; 1998 p. 189–197.

[47] Gordon AL. Brazil-Malvinas Confluence – 1984. Deep-Sea Res. 1989; 3(36): 359–384.

[48] Piola AR, Matano RP, Palma E, Möller OO, Campos EJD. The influence of the Plata river discharge on the western South Atlantic Shelf. Geophys Res Lett. 2005; 32(1): 1–4.

[49] Souza RB, Robinson IS. Lagrangian and satellite observations of the Brazilian Coastal Current. Cont Shelf Res. 2004; 24: 241–262.

[50] Geraci JR, Lounsbury VJ. Specimen and Data Collection In Geraci JR, Lounsbury VJ, editors. Marine mammals ashore: A field guide for strandings 2nd ed. Maryland: National Aquarium in Baltimore; 2005.

[51] Wood SN. Generalized Additive Models An Introduction with R. Boca Raton, FL: Chapman and Hall/CRC; 2006.

[52] Zuur AF, Saveliev AA, Ieno EN. A Beginner’s Guide to Generalised Additive Mixed Models with R. 1st Ed. United Kingdom: Highland Statistic; 2014.

[53] R Development Core Team. R: A language and environment for statistical computing R Foundation for Statistical Computing Vienna, Austria 2008 ISBN 3-900051-07-0, Available: http://www.R-project.org/.

[54] Evans K, Thresher R, Warneke RM, Bradshaw CJA, Pook M, Thiele D, et al Periodic variability in cetacean strandings: links to large-scale climate events. Biol Lett. 2005; 1: 147–150. 17148151 10.1098/rsbl.2005.0313 PMC1626231

[55] Grémillet D, Lewis S, Drapeau L, Lingen CDVD, Huggett JA, Coetzee JC, et al Spatial match-mismatch in the Benguela upwelling zone: should we expect chlorophyll and sea-surface temperature to predict marine predator distribution? J. Appl. Ecol. 2008; 45: 610–621.

[56] Haimovici M, Martins AS, Figueiredo JL, and Vieira PC. Demersal bony fish of the outer shelf and upper slope off southern Brazil subtropical convergence ecosystem. Mar Ecol-Prog Ser. 1994;108:59–77.

[57] Castello JP, Haimovici M, Odebrecht C and Vooren CM. A Plataforma e o Talude Continental In Seeliger U, Odebrecht C, Castello JP, editors. Os ecosistemas costeiro e marinho do extremo sul do Brasil. Rio Grande: Ecoscientia; 1998 p. 189–197.

[58] Pinedo MC, Lammardo MP, Barreto AS. Review of *Ziphius cavirostris*, *Mesoplodon grayi* and *Lagenodelphis hosei* (Cetacea: Ziphiidae and Delphinidae) in Brazilian waters, with new records from southern Brazil. Atlântica. 2001; 23: 67–76.

[59] Santos RA, Haimovici M. Cephalopods in the diet of marine mammals stranded or incidentally caught along southeastern and southern Brazil (21–34°S). Fish Res. 2001; 52: 99–112.

[60] Santos RA, Haimovici M. Cephalopods in the trophic relations off southern Brazil. B Mar Sci. 2002; 71(2): 753–770.

[61] Secchi ER, Ott PH, Crespo OEA, Kinas PG, Pedraza SN, Bordino P. A first estimate of franciscana (*Pontoporia blainvillei*) abundance off southern Brazil. J Cetacean Res Manag. 2001; 3: 95–100.

[62] Danilewicz D, Secchi ER, Ott PH, Moreno IB, Bassoi M, Martins MB. Habitat use patterns of franciscana dolphins (*Pontoporia blainvillei*) off southern Brazil in relation to water depth. J Mar Biol Assoc UK. 2009; 89(5): 943–949.

[63] Di Tullio JC, Fruet PF, Secchi ER. Indentifying critical areas to reduce bycatch of coastal common bottlenose dolphins *Tursiops truncatus* in artisanal fisheries of the subtropical western South Atlantic. End. Spe. Res. 10.3354/esr00698

[64] Reeves RR, Dalebout ML, Jefferson TA, Karczmarski L, Laidre K, O’Corry-Crowe G, et al. *Pontoporia blainvillei* in: IUCN 2015. *IUCN* Red List of Threatened Species. 2008. Version 2012.1. Available: www.iucnredlist.org. Accessed 12 March 2015.

[65] Túnez JI, Cappozzo HL, Cassini MH. Natural and anthropogenic factors associated with the distribution of South American sea lion along the Atlantic coast. Hydrobiologia. 2008; 598: 191–202.

[66] Ferreira JM, De Oliveira LR, Wynen L, Bester MN, Guinet C, Moraes-Barros N, et al Multiple origins of vagrant Subantarctic fur seals: a long journey to the Brazilian coast detected by molecular markers. Polar Biol. 2008; 31(3): 303–308.

[67] Oliveira LR. Variação geográfica do lobo-marinho sulamericano, *Arctocephalus australis* (Zimmermann, 1783) com base em dados morfológicos e moleculares. PhD. Thesis, Universidade de São Paulo. 2004.

[68] Artico LDO, Bianchini A, Grubel KS, Monteiro DDS, Estima SC, De Oliveira, et al Mitochondrial control region haplotypes of the South American sea lion *Otaria flavescens* (Shaw, 1800). Braz J Med Biol Res. 2010; 43(9): 816–820. 20838754 10.1590/s0100-879x2010007500074

[69] Piola AR, Campos EJD, Möller JOO, Charo M, Martinez CM. Subtropical shelf front off eastern South America. J. Geophys. Res. 2000; 105(C3): 6566–6578.

[70] Garrigue C, Ross GJB. A record of a subantarctic fur seal, *Arctocephalus tropicalis*, from Madagascar, Indian Ocean. Mar Mammal Sci. 1996; 125: 624–627.

[71] David JHM, Salmon L. Records of the subantarctic fur seal from Rodrigues and Mauritius, Indian Ocean. Afr J Mar Sci. 2003; 25: 403–405.

[72] Hofmeyr GJG, Amir OA. Vagrant Subantarctic fur seal on the coast of Tanzania. Afr. Zool. 2010; 45(1): 144–146.

[73] Rosas FCW, Pinedo MC, Marmontel M, Haimovici M. Seasonal movements of South American sea lion (*Otaria flavescens*, Shaw) off the Rio Grande do Sul coast, Brazil. Mammalia. 1994; 58(1): 51–59.

[74] Pavanato H, Silva KG, Estima SC, Monteiro DS, Kinas PG. Occupancy dynamics of South American Sea-Lions in Brazilian Haul-outs. Braz J Biol. 2013; 73(4): 855–862. 24789403 10.1590/s1519-69842013000400023

[75] Páez E. Situacion de la administracion del recurso lobos y leones marinos en Uruguay In Menafra R, Rodriguez-Gallego L, Scarabino F, Conde D, editors. Bases para la conservacion y el manejo de la costa uruguaya. Uruguay: Vida Silvestre; 2006 p. 577–583.

[76] Páez E. Dinámica de la población de hembras de lobo fino sudamericano *(Arctocephalus australis)* en Uruguay In Gutlérrez N, Defeo O, editors. Evaluación de recursos pesqueros de Uruguay mediante modelos dinâmicos. Proyecto Gestión Pesquera en Uruguay. Montevideo: MGAP-DINARA–FAO; 2013 p. 65–78.

[77] Crespo EA, Oliva D, Dans SL, Sepúlveda M. 2012. Current status of the South American sea lion along the distribution range Valparaíso: Universidad de Valparaíso Press; 2012.

[78] Machado R, Oliveira LR, Montealegre-Quijano S. Incidental catch of South American sea lion in a pair trawl off southern Brazil. Neot Biol Conserv. 2015; 10(1): 43–47.

[79] Pinedo MC. Ocorrência de Pinípedes na costa brasileira. Garcia de Orla, Série Zoologia. 1990; 15(2): 37–48.

[80] Oliveira LR. Caracterização dos padrões de ocorrências de pinípedes (Carnivora, Pinnipedia) ocorrentes no litoral norte do estado do Rio Grande do Sul, Brasil, entre abril de 1993 e dezembro de 1998. M.Sc. Thesis, Pontifícia Universidade Católica do Rio Grande do Sul. 1999.

[81] FIiedler PC. Environmental change in the eastern tropical Pacific Ocean: a review of ENSO and decadal variability. Mar Ecol Prog Ser. 2002; 244: 265–283.

[82] Trathan PN, Forcada J, Murphy EJ. Environmental forcing and Southern Ocean marine predator populations: effects of climate change and variability. Phil trans R Soc. 2007; 362: 2351–2365. 10.1098/rstb.2006.1953 PMC2443178 17553770

[83] Forcada J, Trathan PN, Reid K, Murphy EJ, Croxall JP. Contrasting population changes in sympatric penguin species with climate warming. Glob Change Biol. 2006; 12: 411–423.

[84] Lea MA, Guinet C, Cherel Y, Duhamel G, Dubroca L, Pruvost P, et al Impacts of climatic anomalies on provisioning strategies of a Southern Ocean predator. Mar Ecol-Prog Ser. 2006; 310: 77–94.

[85] Moura JF, Siciliano S. Straggler Subantarctic Fur Seals (*Arctocephalus tropicalis*) on the coast of Rio de Janeiro state, Brazil. Lat Am J Aquat Mamm. 2007; 6(1): 103–107.

[86] Zerbini AN, Kotas JE. A note on cetacean bycatch in pelagic driftnetting off Southern Brazil. Rep Int Whal Commn. 1998; 48: 519–524.

[87] Dalla Rosa L, Secchi ER. Killer whale (*Orcinus orca*) interactions with the tuna and swordfish longline fishery off southern and south-eastern Brazil: a comparison with shark interactions. J Mar Biol Assoc UK. 2007; 87: 135–140.

[88] Passadore C, Domingo A, Secchi ER. Analysis of marine mammal bycatch in the Uruguayan pelagic longline fishery operating in the Southwestern Atlantic Ocean. ICES J Mar Sci. 2015; 72: 1637–1652.

[89] Secchi ER Jr. Vaske T. Killer whale (*Orcinus orca*) sightings and depredation on tuna and swordfish longline catches in southern Brazil. Aquat Mamm. 1998; 24: 117–122.

[90] Zerbini AN, Secchi ER, Bassoi M, Dalla-Rosa L, Higa A, Sousa LD, et al Distribuição e abundância relativa de cetáceos na Zona Econômica Exclusiva da região sudeste-sul do Brasil Série de Documentos Revizee-Score Sul. São Paulo: Instituto Oceanográfico-USP; 2004.

[91] Passadore C, Domingo A, Secchi ER. Depretion by killer whale (*Orcinus orca*) and false killer whale (*Pseudorca crassidens*) on the catch of the Uruguayan pelagic longline fishery in Southwestern Atlantic Ocean. ICES J Mar Sci. 2015 10.1093/icesjms/fsu251

[92] Botta S, Hohn AA, Nacko AS, Secchi ER. Isotopic variation in delphinids from the subtropical western South Atlantic. J Mar Biol Assoc UK. 2012; 92(8): 168–1698.

[93] Lodi L, Hetzel B. O golfinho-de-dentes-rugosos (*Steno bredanensis*) no Brasil. Bioikos. 1998; 12(1): 29–45.

[94] Groch KR, Palazzo JT, Flores PA, Adler FR, Fabian ME. Recent rapid increases in right whale (*Eubalaena australis*) population off southern Brazil. Lat Am J Aquat Mamm. 2005; 4(1): 41–47.

[95] Ward E, Zerbini AN, Kinas PG, Engel MH, Andriolo A. Estimates of population growth rates of humpback whales (*Megaptera novaeangliae*) in the wintering grounds off the coast of Brazil (Breeding Stock A). J Cetacean Res Manag. 2011; 3: 145–149.

[96] Castello HP, Pinedo MC. Southern right whales(*Eubalaena australis*) along the southern Brazilian coast. J Mammal. 1979;60(2): 429–430.

[97] Seyboth E, Groch KR, Secchi ER, Dalla Rosa L. Habitat use by southern right whales, *Eubalaena australis* (Desmoulins, 1822), in their main northernmost calving area in the western South Atlantic. Mar Mammal Sci. 2015 10.1111/mms.12241

[98] Greig AB, Secchi ER, Zerbini AN, Dalla Rosa L. Stranding events of southern right whales, *Eubalaena australis*, in southern Brazil. J Cetacean Res Manag. 2001; 2: 157–160.

[99] Zerbini AN, Andriolo A, Heide-Jørgensen MP, Pizzorno JL, Maia YG, VanBlaricom GR, et al Satellite-monitored movements of humpback whales (*Megaptera novaeangliae*) in the Southwest Atlantic Ocean. Mar Ecol-Prog Ser. 2006; 313: 295–304.

[100] Zerbini AN, Andriolo A, Heide-Jørgensen MP, Moreira SC, Pizzorno JL, Maia YG, et al Migration and summer destinations of humpback whales (*Megaptera novaeangliae*) in the western South Atlantic Ocean. J Cetacean Res Manag. (Special issue). 2011; 3: 113–118.

[101] Zerbini AN, Secchi ER, Siciliano S, Simões-Lopes PC. A review of the occurrence and distribution of whales of the genus Balaenoptera along the Brazilian coast. Rep Int Whal Commn. 1997; 47: 407–417.

[102] Hassel LB, Venturotti A, Magalhães FA, Cuenca S, Siciliano S, Marques FFC. Summer sightings of dwarf minke whale (*Balaenoptera acutorostrata*) off the eastern coast of Rio de Janeiro state, Brazil. Lat Am J Aquat Mamm. 2003; 2(1): 47–50.

[103] Kawamura A. A review of food in balaenopterid whales. Scientific Reports of the Whales Research Institute. 1980; 32: 155–197.

[104] Bannister JL. Baleen Whales (Mysticetes) In Perrin WF, Wursig B, Thewissen JGM, editors. Encyclopedia of marine mammals. San Diego: Academic Press; 2009 p. 80–89.

[105] Moura JF, Siciliano S. Stranding pattern of Bryde’s whales along the south-eastern coast of Brazil. Mar. Biodivers. Rec. 2012; 5: 1–7.

[106] Wojtek B, Norman SA. *Ziphius cavirostris* strandings-a short review.Smithsonian. 2010; 1000(229): 13–07.

[107] Toledo GAC, Gurgel Filho NM, Brito JLC, Langguth A. Stranding of a Risso’s dolphin (Cetacea, Delphinidae) on the north-eastern coast of Brazil, with comments on its distribution and threats in the Western South Atlantic. Mar Biodivers Rec. 2015; 8: 1–7.

[108] Lumpkin RS, Garzoli S. Interannual to decadal changes in the western South Atlantic’s surface circulation. J Geophys Res. 2011; 116: 1–10.

