# Supplementary figures and images for: Long-Term Seasonal and Interannual Patterns of Marine Mammal Strandings in Subtropical Western South Atlantic
Source: PLoS One. 2016 Jan 27;11(1):e0146339. doi: 10.1371/journal.pone.0146339 (PMC4729480; doi:10.1371/journal.pone.0146339)

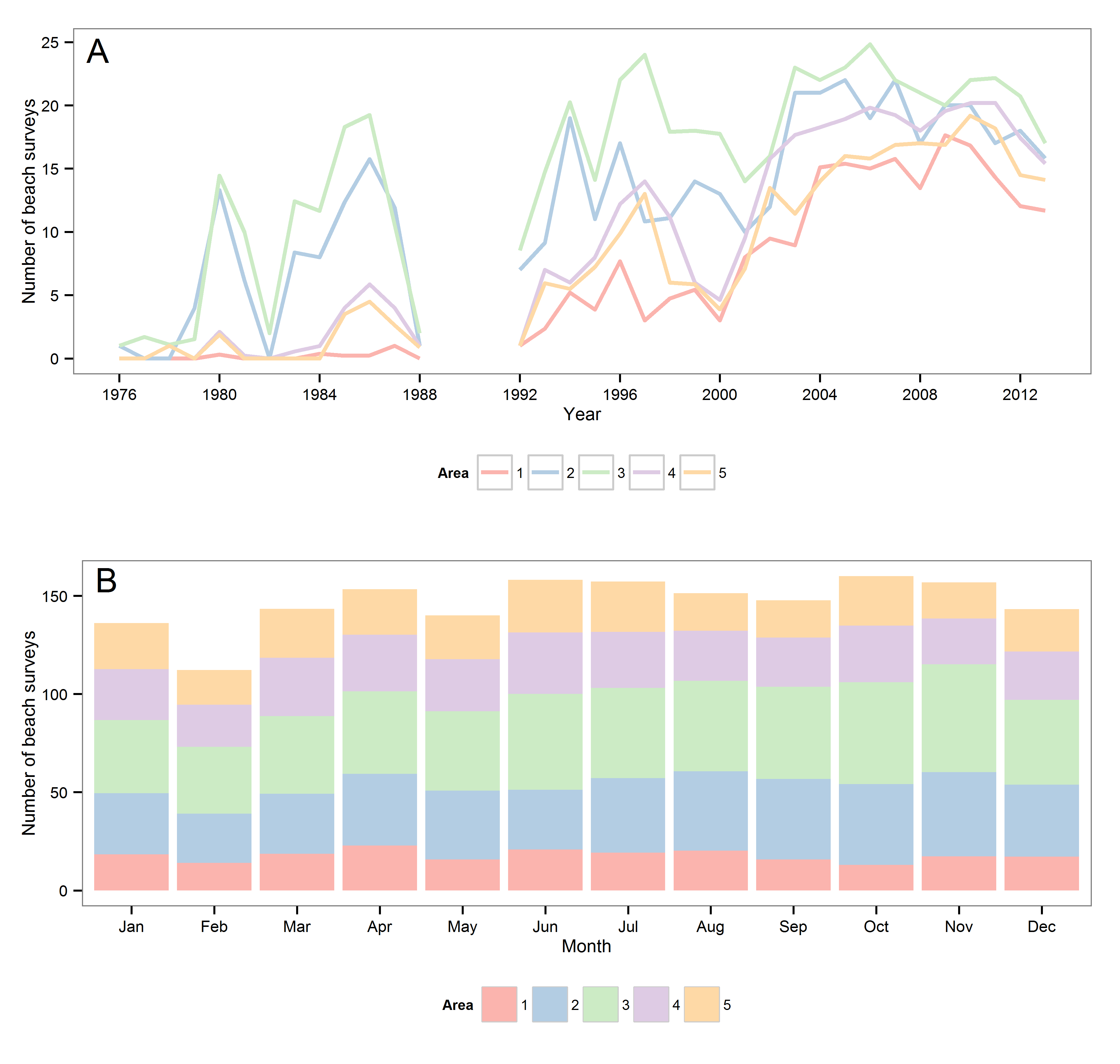

Supplement: S1 Fig — I = 84km; II = 51km; III = 63km; IV = 70km; V = 87km. (TIFF) [file pone.0146339.s001.tiff]
