# Supplementary material for: Long-Term Seasonal and Interannual Patterns of Marine Mammal Strandings in Subtropical Western South Atlantic
Source: PLoS One. 2016 Jan 27;11(1):e0146339. doi: 10.1371/journal.pone.0146339 (PMC4729480; doi:10.1371/journal.pone.0146339)
Supplement: S1 Table — (DOCX) [file pone.0146339.s002.docx]

| **Year** | **Month** | **Species** | **No. of animals** |
| --- | --- | --- | --- |
| **1993** | December | *Pseudorca crassidens* | 2 |
| **1995** | June | *Pseudorca crassidens* | 14 |
| **1997** | November | *Lagenodelphis hosei* | 4 |
| **1998** | September | *Pseudorca crassidens* | 2 |
| **2009** | January | *Physeter macrocephalus* | 4 |
| **2011** | February | *Steno bredanensis* | 4 |
| **2012** | January | *Physeter macrocephalus* | 2 |

**S1 Table. Marine mammal mass strandings (*n*=7) during 1976–2013 in southern Brazil.**
